# Retraction Note: Short-time QiBaoMeiRan Formula Treatment Exerts Estrogenic Activities without Side Effects on Reproductive Tissues in Immature Mice

**DOI:** 10.1038/s41598-021-92516-1

**Published:** 2021-06-22

**Authors:** Ying Xu, Xiao-ping Ma, Jin-na An, Zi-jia Zhang, Jie Ding, Ya-kun Qu, Zhen-li Liu, Na Lin

**Affiliations:** 1 Institute of Chinese Materia Medica, China Academy of Chinese Medical Sciences, Dongcheng District Dongzhimen Nanxiao Road 16, Beijing, 100700 China; 2 The MOE Key Laboratory for Standardization of Chinese Medicines and The Key Laboratory for Pharmacology of Compound Chinese Medicine of Shanghai, Institute of Chinese Materia Medica, Shanghai University of Traditional Chinese Medicine, Shanghai, China; 3 Institute of Basic Theory, China Academy of Chinese Medical Sciences, Dongcheng District Dongzhimen Nanxiao Road 16, Beijing, 100700 China

Retraction of: *Scientific Reports* 10.1038/srep17436, published online 08 December 2015

The Editors have retracted this Article.

After publication, concerns were raised with respect to some of the data reported. Specifically:

- In Figure 2 the panels ii and iv of (B) and the ERβ and PCNA slides in row ii of (C) appear to contain internal duplications;

- The GAPDH loading controls in Figure 2D and Figure 6 appear to be the same.

The Authors provided original data files for all figures except Figure 6. Examination of the files for Figure 2B and 2C could not exclude that the duplications were image acquisition artefacts. However, when the original data files for Figure 1D and 2D were examined evidence of the removal of background noise and non-specific signal was apparent, in violation of the Journal’s policy on digital integrity and standards.

The Editors therefore no longer have confidence in the data presented.

Na Lin and Ying Xu did not explicitly state whether they agree to this retraction notice. None of the other co-authors responded to the correspondence about this retraction.

